# Embedding interpersonal stigma resistance into the medical curriculum: a focus group study of medical students

**DOI:** 10.1186/s12909-023-04512-w

**Published:** 2023-09-21

**Authors:** Ashley McAllister, Kara Dickson, Mediya Rangi, Leonie Griffiths, Stefanie Dimov, Nicola Reavley, Stephanie Knaak

**Affiliations:** 1 School of Population and Global Health, The University of Melbourne, Level 4, 207 Bouverie Street, Carlton, VIC 3053 Australia; 2 Melbourne Medical School, The University of Melbourne, Level 4, 207 Bouverie Street, Carlton, VIC 3053 Australia; 3 Faculty of Nursing and Department of Psychiatry, University of Calgary, 2500 University Drive NW, Calgary, Alberta T2N 1N4 Canada

**Keywords:** Qualitative research, Focus groups, Mental health, Stigma, Stigma reduction, Curriculum design, Student perceptions, Healthcare

## Abstract

**Background:**

Mental-health-related stigma among physicians towards people with mental illnesses remains a barrier to quality care, yet few curricula provide training with a proactive focus to reduce the potential negative impacts of stigma. The aim of our study was to explore medical students’ perspectives on what areas of learning should be targeted (where stigma presents) and how they could be supported to prevent the formation of negative attitudes.

**Methods:**

Six focus group discussions were conducted with second, third, and fourth-year postgraduate medical students (*n* = 34) enrolled at The University of Melbourne Medical School in September – October 2021. Transcripts were analysed using inductive thematic analysis.

**Results:**

In terms of where stigma presents, three main themes emerged – (1) through unpreparedness in dealing with patients with mental health conditions, (2) noticing mentors expressing stigma and (3) through the culture of medicine. The primary theme related to 'how best to support students to prevent negative attitudes from forming' was building stigma resistance to reduce the likelihood of perpetuating stigma towards patients with mental health conditions and therefore enhance patient care. The participants suggest six primary techniques to build stigma resistance, including (1) reflection, (2) skills building, (3) patient experiences, (4) examples and exemplars, (5) clinical application and (6) transforming structural barriers. We suggest these techniques combine to form the ReSPECT model for stigma resistance in the curriculum.

**Conclusions:**

The ReSPECT model derived from our research could provide a blueprint for medical educators to integrate stigma resistance throughout the curriculum from year one to better equip medical students with the potential to reduce interpersonal stigma and perhaps self-stigma. Ultimately, building stigma resistance could enhance care towards patients with mental health conditions and hopefully improve patient outcomes.

## Background

Most physicians will work with patients needing mental health support throughout their careers. However, stigma among physicians towards people with mental health conditions remains a barrier to quality care [1, 2], yet few curricula provide training with a proactive focus to reduce the potential negative impacts of stigma [3, 4].

### What is mental health-related stigma and its impact?

Mental health-related stigma (hereto referred to as stigma) is when a person is perceived and/or treated in a negative way because of their mental health condition [5]. Evidence shows several types of stigma including at the individual, interpersonal and structural level [5]. In this paper, we focus on interpersonal stigma. More specifically, we focus on interpersonal stigma related to physicians’ attitudes and behaviour. Interpersonal stigma (also known as public stigma) refers to the attribution of knowledge (often stereotypes or misinformation), negative attitudes (prejudice) and/or negative behaviours (discrimination) towards people with mental health conditions as a result of their condition [6, 7]. In effect, it is the interactions that can occur between the stigmatised and the non-stigmatised [8].

Many patients report experiences of interpersonal stigma amongst health practitioners [1, 9–13]. It can manifest in several ways including diagnostic and treatment overshadowing (where a health professional attributes a person’s physical symptoms to their mental health condition [14]), non-caring and unhelpful behaviours, excluding and rejecting people from health services, and coercive practices [15, 16]. Furthermore, people with mental health conditions may avoid seeking mental health support due to the widespread stigma that exists among health professionals [1]. Inattention to the problem of stigma among health providers can lead to poorer quality healthcare and worse health outcomes for people with mental health conditions [17]. For example, people with complex mental health conditions die earlier than the general population, with an average estimated 10–25-year life expectancy gap between these groups – a gap that has widened in Australia in recent years [18]. It is increasingly believed that this life expectancy gap will not shift until the many types of stigma that people with complex mental health conditions encounter are sufficiently addressed, in particular diagnostic overshadowing [2, 19, 20].

### Why target medical students?

Medical students are the next generation of physicians. These students will enter the workforce and become the agents of change needed to reduce stigma and improve patient care. Tackling stigma early in a health practitioner’s career is a crucial way of addressing stigma later in practice. It is a time when students are more sensitised to role models and developing their own professional identity. Several programs have demonstrated their success with improving the attitudes of medical and nursing students toward working with people with lived experience of mental health conditions (e.g., [4, 21]). Most interventions that show a decrease in negative attitudes are contact based (e.g., include learning experiences with people with lived experience of mental health conditions) (e.g., [22]) or education-based (e.g., presenting factual information about mental health conditions with the intention of correcting misinformation or improving mental health literacy) (e.g., [23]) or a combination of these two types (e.g., [4, 24, 25]). However, many interventions are short-term, one-off and rarely embedded into health professional curricula. This approach to reducing stigma may not be long lasting. As a result, The Australian Productivity Commission on Mental Health recommended embedding stigma reduction into health professional education (Action 16.6) [26]. This ensures consistent exposure and learning opportunities and signals to students that the topic is not tokenistic, increasing their engagement and attention to the subject matter. Yet, limited evidence exists on how this might be achieved. This study addresses this gap by seeking medical students’ input on how addressing stigma might be embedded into curricula. Involving medical students in curriculum development provides insight into addressing stigma challenges, leading to tailored interventions. In addition, medical students’ contribution to developing these interventions could create a sense of ownership, and investment may increase the application of learning in future clinical practice [27].

### The University of Melbourne Medical School

The Doctor of Medicine is a four-year course designed to offer a flexible education model. Students undertake clinical placements in hospital and primary care from the commencement of the course to develop their person-centred communication skills and integrate bioscience and population health into their early practice. In the second year, students learn about The Mental State Examination. During the third year, students undertake a six-week Mental Health rotation introducing them to inpatient and outpatient psychiatry. Finally, they consolidate their application of the Mental State Examination and receive teaching on the value of learning from people with lived experience of mental health conditions.

Furthermore, the third year of the course includes a six-week rotation in General Practice whereby students will consult with patients who may have mental health conditions. In the final year of the course, students may undertake studies in psychiatry through the elective rotation. Finally, Professional Practice tutorials are embedded throughout all four years of the course, allowing students to reflect on their experiences interacting with patients. Examples of tutorial topics include challenging stereotypes and stigma in the context of patients who have diverse abilities and learning graded assertiveness.

### Study aims

The aim of this study was to explore from the perspective of medical students how to best embed stigma reduction into medical curriculum. We focused on two main research questions:

1. What areas of learning should we target (where does stigma present)?
2. How could medical students be supported to prevent negative attitudes from forming?

## Methods

### Methodology

In this study, we drew on ethnographic methodology and used a key informant approach [28] employing ethnographic focus group discussions (FGD) as the main data collection technique. In this study, key informants were medical students as we wanted to explore solutions for students by students. FGDs provide a unique opportunity to collectively interview participants and observe them while interacting [29]. Furthermore, the advantages of FGDs include their ability to facilitate interaction and give access to informants' attitudes and experiences [29]. The design of ethnographic FGDs means collecting participants' stories in a feasible yet unobtrusive way [30].

We used the Consolidated Criteria for Reporting Qualitative Research (COREQ) checklist to design and report findings from this study [31].

### Study participants

Study participants were recruited from the University of Melbourne Medical School in Melbourne, Australia. Purposive sampling was used to identify participants and inclusion criteria were students enrolled in second-, third- or fourth year of medical school. No further selection criteria were applied. Participants were compensated for their time with a $50 Visa gift voucher.

An email invitation was sent via a central mailing system to all enrolled second, third- and fourth-year medical students at the University of Melbourne (UoM) (approximately 1,067 students). The email was sent twice over a two-week period. 122 students registered interest in the study. Participants were selected to participate based on a first come, first serve basis but the consideration for diversity within each focus group was taken (e.g., equal gender, year levels and clinical school).

Six FGDs were conducted online via Zoom in September to October 2021. Thirty-four students participated with three to seven students in each focus group. Each focus group contained a mix of year levels, gender and clinical schools and lasted between 69 and 78 min. Table 1 summarises participant characteristics.

**Table 1:** Participant characteristics

Characteristic		N (%)
Number of participants		34
% female		17 (50%)
Mean age (years, min–max)		24.6 (19–31)
Year of medical school (n)	MD2	11 (32%)
	MD3	14 (41%)
	MD4	9 (26%)
% who have commenced or completed a clinical placement in mental health		20 (59%)

### Setting

Ash McAllister (AM), a female, non-medical senior research fellow with extensive experience in qualitative research, facilitated all FGDs, MR co-facilitated five FGDs and SD co-facilitated one. Both co-facilitators were female, non-medically trained researchers working in a public health research unit. The role of the co-facilitator was to take field notes and monitor participant distress. There were no non-participants present in the FGDs. Demographic information including age, gender, year level, clinical school and whether the student had completed their mental health rotation was collected. The facilitator initiated the FGDs with a preamble which included the impetus for the study, assumptions (i.e., the literature shows that stigma does exist in health care settings) and how the findings of the study would be used.

### Data collection

Prior to the study, an unstructured topic guide was developed by AM and reviewed by all co-authors for input. Each focus group began with an introduction of the project and brief definitions of mental-health related interpersonal stigma. It was also emphasised that we were interested in stigma related to less common mental health conditions (e.g., schizophrenia, bipolar disorder) as literature suggests these conditions have seen the least improvement in stigma reduction [32]. The aims of the FGDs were to explore two main topics: (1) where stigma presents in medical education including clinical placements, and (2) how students could be supported to prevent negative attitudes from forming. Examples of questions from the FGD guide included:

- In your experience, where does stigma typically show up?
- Evidence shows that clinical placements are a transition time for attitudes. How could you be supported to avoid negative attitudes towards people with mental health conditions?
- What would motivate you to participate in an anti-stigma intervention?

Participants received an email with the participant information statement, a link to complete the consent form and examples of broad topics prior to the focus group. No repeat FGDs were conducted. All FGDs were audio recorded and professionally transcribed. AM, MR and SD took fieldnotes during the FGDs and AM wrote separate fieldnotes after each focus group reflecting on key points and connecting themes between each FDG. AM and MR discussed data saturation after the fourth focus group and determined that the six planned FGDs were sufficient to capture emerging themes and no further groups were required.

### Data analysis

AM and KD were the primary coders of the transcripts. However, LG coded the first two transcripts and had analysis debriefings with AM. LG’s clinical background provided a unique perspective and the additional coding was completed to ensure that contextual factors were not overlooked. An inductive thematic analysis approach was used following Braun and Clarke’s [33] six steps for thematic analysis. Delve software was used to code transcripts. The first two transcripts were coded line by line and then discussed among the research team. Parent codes were developed, and the remaining transcripts were coded using an iterative process to review, define and refine codes. In this paper, the codes relevant to where stigma presents and how to support students in preventing negative attitudes from forming are presented.

### Ethics

Study participation was voluntary. All students were informed about the purpose of the study, anonymity and confidentiality of responses. All participants provided written informed e-consent. Ethics approval was granted by The University of Melbourne Office of Research Ethics and Integrity (Project ID: 2021–21,542).

## Results

We present the results as answering the two main research questions (1) what areas of learning should we target (where does stigma present)? and (2) how could medical students be supported to prevent negative attitudes from forming?

Participants spoke about three main areas where stigma presents – (1) through unpreparedness in dealing with patients with mental health conditions (2) noticing mentors expressing stigma and (3) through the culture of medicine. In terms of the second research question, “how can medical students be supported to prevent negative attitudes from forming?”, the primary theme that emerged from the FGDs was that of building stigma resistance. The following section elaborates on findings. To delineate between participants, MD(Year) denotes the year the participant is in i.e., MD4 denotes a fourth-year medical student, and FG# denotes what focus group they were in i.e., FG3 will denote focus group three.

### Where stigma presents

#### Feeling unprepared to manage patients with mental health conditions

Inadequate mental health training and knowledge was considered a key driver of stigma. Participants indicated that they encountered patients with mental health conditions in all clinical settings and largely felt unprepared, and “totally unequipped” (MD4, FG6) to respond to patient’s concerns:

“S*tigma essentially comes from a lack of education and exposure.” (MD4, FG5)*

*“…the very first patient I saw this year on psych rotation was like seven in the morning, first day I was really nervous, I walked in and I went, “Hey, how are you?” And he went, “Well I want to kill myself,” and I went, oh right, here we go, and I had no idea what to do.”* MD4, FG5

The current curriculum structure, which includes minimal mental health education until primarily third year, was identified as a key issue in this regard.

A lack of opportunity for clinical debriefing of difficult patient situations was identified as another way partipants felt ill-equipped to work with patients with mental health conditions.

“*There’s just no space to have those discussions”* (MD2, FG5).

Participants described various ways in which this lack of training and debrefiing led to stigma, including a desire for avoidance, and shifting the responsibility for managing patients with mental health conditions to others, which further perpetuates lack of exposure and stigma.

*“…Mental health is something that comes up in every rotation and you feel under prepared, and you try and avoid it because you haven’t been taught.”* MD3, FG2

#### Noticing mentors expressing stigma

Participants emphasized the importance of role models in influencing the way they approached their practice and adopted certain attitudes. As one participant expressed,

*“we find it really difficult to interact with patients and we’re really influenced by the older medical students and the physicians that we’re following around because that’s the only training that we get until we do a psych rotation”* (MD2, FG5).

Students encountered positive and negative examples of clinical practice on placements. While several participants described positive impacts on patient care and attitudes as a result of being influenced by clinicians who approach mental health from *“a much more educated and sensitive point of view”* (MD4, FG4), many participants also discussed observing mentors expressing stigma in clinical settings, formally and informally, and provided examples of stigma expressed by all types of health care professionals at all stages of their career.

Participants provided several examples where they witnessed stigmatising language about patients, dismissing patients, and even threats to refuse care. This seemed to be particularly the case for conditions such as eating disorders, personality disorders and substance use which carry the perception of being complex and difficult to treat.

*“And before the patient was put under, they started referring to the patient as a slasher to their face. And then, throughout the rest of the surgery, just referred to the patient as the slasher-this, slasher-that, slasher-that, and none of the other surgeons or anyone seemed to pick up on it, or even confront it.”* MD2, FG4

*“Gen-med, put simply, just does not want to deal with them [eating disorders],…very much, yeah, like battling to get them discharged, even though they’re not medically stable…And I’ve seen that on multiple teams now, it’s not just the one or two registrars, or consultants, it seems to be quite prolific throughout.” MD3, FG4*

*“The way physicians have talked about people who do have borderline personality, they’ll be, like, “Oh, they’re showing personality traits,” or, “Oh, it’s just another overdose, we just need to get them out.”* MD3, FG2

In general, participants felt that the level or degree of stigma they observed tended to vary between specialities. Many indicated noticing mentors expressing negative attitudes and behaviours most in the ED and surgery. In one focus group, participants said they noticed less stigma in ‘lifestyle specialities’ such as ophthalmology, general practice and dermatology. The rationale was that because these specialities often afforded more time to build relationships with patients, these physicians might be more aware of the role of social determinants of health compared to some other specialties, and these fields might also provide more work-life balance and therefore a less toxic culture.

#### Culture of medicine

All FGDs described how the culture of medicine can enable stigma. However, participants spoke about this relationship in different ways. For many, it was about the hierarchical nature of medicine.

*“The hierarchy of medicine. I mean, look, medicine is so hierarchical, it is ridiculously hierarchical and combating stigma within a hierarchical system is hugely – all graded assertiveness within a hierarchical system is hugely challenging.”* MD4, FG1

It was in this context that the challenge of speaking up when witnessing stigma was raised. In all of the FGDs, participants described feeling uncomfortable and at times distressed by having witnessed stigma but feeling ill-equipped and powerless to speak up due to the hierarchical nature of the medical system.

*“[Witnessing stigma] made [students] feel really horrible about it, and they wanted to help that patient, but it's not appropriate as a medical student to step in and disagree with a clinician.”* MD3, FG3

*“There’s a massive power imbalance for us, and it shames me to say it… but if you kind of don’t get on well with them then there’s a good chance that you’ll miss out on learning what you need to learn.”* MD2, FG6

*“…if people above you who can, to some extent, determine your professional life, us speaking about stereotypes you can’t really, again, really bear to voice your opinions.”* MD3, FG6

Participants also noted that while their curriculum teaches them graded assertiveness, the staunch hierarchy of the culture of medicine makes this very difficult to put into practice.

While self-stigma was not part of the interview guide, participants in each FGD nevertheless raised it as a point of discussion. In particular, they spoke of the prevalence of self-stigma among physicians, and discussed the strongly ingrained cultural belief that physicians are not supposed to have mental health issues, that prioritising self-care is perceived as a sign of weakness, and that taking time off or disclosing mental health issues is to be avoided due to fear of adverse impacts on career progression.

*“It’s okay, to not be doing okay, but physicians seem to have this stigma that no, you have to handle it, you have to cope.”* MD4, FG1

*“I think a lot of times we have to appear to be competent, which comes with the implied notation that we can’t have mental health issues.”* MD3, FG6

Notably, several participants linked this issue of culture and self-stigma to the expression of interpersonal stigma towards patients with mental health conditions:

*“There’s this stigma and expectation that we should be immune to it, that physicians aren’t allowed to have mental health problems… I think if we don’t learn to talk about ourselves… how are we going to be able to do good for our patients?”* MD4, FG1

Another way the culture of medicine seems to perpetuate stigma is through the stereotypes attached to the profession of psychiatry. Several participants provided examples in this regard.

*“A lot of other physicians make comments about it [psychiatry] not being a real medical profession and that they’d never want to do it because there are no real outcomes...”* MD4, FG4

“*I think a lot of the stigma that I’ve seen over the years is aimed at psychiatrists themselves, and sort of the field in general.”* MD4, FG4

*“I’ve heard one, you know you’re in a psych consult when you can’t tell who’s the patient and who’s the physician.”* MD3, FG1

While participants also provided examples of efforts towards cultural change, including curriculum activities such as R U OK Day and some mindfulness activities, the consensus remained that that there needs to be considerably greater focus on culture change within the medical profession.

### How students can be supported – building interpersonal stigma resistance

In terms of the second research question, “how can medical students be supported to prevent negative attitudes from forming?”, the primary theme that emerged was that of building stigma resistance, which participants described as the capacity to remain unaffected by mental health-related stigma even when faced with it or witnessing it [33, 34].

**Fig. 1:**
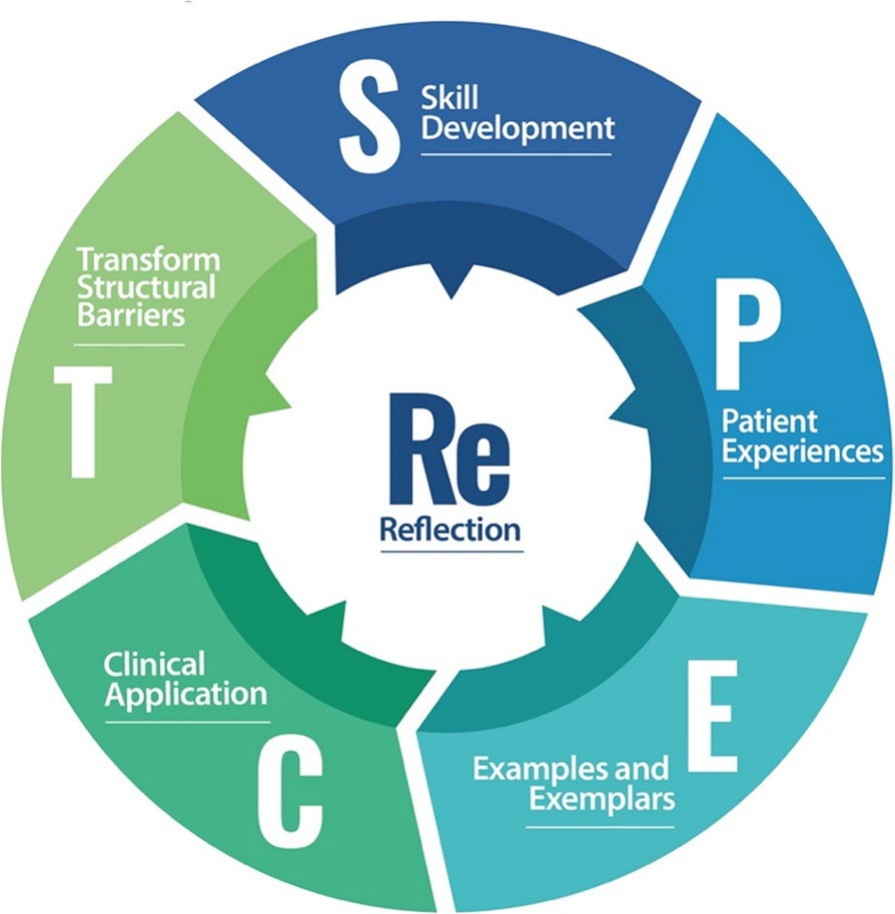
ReSPECT model for interpersonal stigma resistance in curriculum

Participants suggested several techniques to build stigma resistance that would help address the sources of stigma discussed above. These included: (1) reflection, (2) skills development, (3) patient experiences, (4) examples and exemplars, (5) clinical application and (6) transformation of structural barriers. These techniques have been summarized into a conceptual framework called the ‘ReSPECT model for stigma resistance in curriculum’ (Fig. 1), and are described in more detail below.

#### (Critical) reflection

In as much as participants described a lack of formal opportunity for guided reflection and debrief about mental health in the curriculum, participants across all six FGDs emphasized that they searched for opportunitities to debrief about challenging clinical experiences informally:

*I wasn't able to debrief necessarily with the physician very well, because they were in a rush to get through their patients…. And so, I ended up just going to one of my mates who is a physician, I'm like, “I need to call you, I need to debrief with someone. I don't know who to talk to.”* MD2, FG3

In this context, participants felt there was an opportunity for medical educators to facilitate reflection and debriefing in an educational space to better support students and enhance the learning opportunities for *all* students.

For example, some participants suggested that clinical practice tutorials could provide a safe space for facilitated reflection and discussion with a supervisor. This could allow them to reconcile distress and learn from their experiences. Importantly, participants noted that for tutorials to be beneficial, group composition of tutorial participants needs to be selected to ensure that students feel comfortable and safe and facilitated by a skilled tutor.

#### Skills development

When asked what might better prepare students to interact with patients experiencing mental health conditions, most suggestions related to building communication skills, as several participants described anxieties around engaging with patients with mental health conditions where they felt fearful that they may *“trigger someone”* (MD3, FG4) or *“say the wrong thing and make their presentation [of a mental health condition] worse”* (MD3, FG2).

Participants across all FGDs expressed a strong appetite for developing foundational skills in mental health and practicing these skills throughout the duration of the course. As one participant expressed: *“I want to be able to practise and get to a level where I will be comfortable in future taking a history from a patient like that [with a mental health condition]”(*MD3, FG2). Participants wanted to be proactive about learning the skills for situations they will inevitably encounter during their clinical placements. Participants suggested specific tools such as frameworks for conducting case histories, discussions about how to conduct sensitive mental health examinations and discussions about appropriate phrasing and ways to approach people with mental health conditions.

*“Having some standard questions that we could use to help patients unpack mental health issues I think could be really helpful.”* MD2, FG2

Participants noted that a toolkit for how to engage with patients with mental health conditions would be welcomed. Furthermore, when asked if these skills should be mandatory, most participants agreed they should. Medical students are time poor and therefore are unlikely to participate in optional activities.

*“It needs to be mandatory because it demonstrated that the med school’s prioritising this learning.”* MD4, FG4

Making mental health mandatory signifies its value in the curriculum which makes its inclusion seem less tokenistic.

Several participants also emphasised that mental health learning activities should be integrated into the curriculum, and that it should be included from year one.

*“[Mental health and mental health conditions are] obviously a major part of health everywhere…and I don’t know why it isn’t much more talked about from day one because it’s not like it’s something we won’t encounter.”* MD2, FG1

Participants also wanted to develop skills in talking about mental health in general, to all patients, not just those seeking treatment specifically for a mental health condition.

*“I think building that comfort to talk about mental health is a big part of it…but just being able to actually have a conversation, say, one of the things I’d like to know about you is how is your mental health, what do you do to keep mentally healthy, what are you [sic] management strategies, and having those conversations….”* MD4, FG1

Participants noted that learning skills must also take into account the context in which they will apply them. Several participants explained the paradox of spending time with a patient to learn their story but also meeting clinical time pressures. For example, a participant described being reprimanded for taking too much time on a case history as they were trying to build rapport with the patient. Participants felt that having more opportunities to learn and practice communication skills in the curriculum would better prepare them for their clinical placements and reduce stigmatising attitudes and behaviours from developing.

#### Patient experiences

Participants expressed wanting to learn more about patients and their journeys. Several perceived that understanding the life of the patient leads to more compassion and empathy. A few participants provided examples of when they had opportunities to chat with patients in placement or when patients came to teach them as part of their curriculum. It was generally agreed that these opportunities make lasting impressions, cement learning, and reduce negative attitudes.

*“…someone might give me a lecture on schizophrenia or depression, but if I actually find out more about the person... that is a much more meaningful encounter and I’ll remember it, I’ll remember the skills that I learnt from it.”* MD2, FG2

*“...hearing from a patient who's positioned as an expert, who's teaching you about their condition and their experience…it humanises these patients, and it humanises these conditions”*(MD2, FG3).

Participants felt that more exposure to, and learning from, people with mental health conditions would help them to develop more empathy and understanding and prevent them from adopting stigmatising views. Patient colloquiums were identified as an example of an existing contact-based intervention that could be adapted to include patients with mental health conditions. They were considered a highly beneficial learning tool that helps early year medical students to understand a patient’s lived experience with different conditions and to see how these conditions can be effectively managed in a range of acute and non-acute settings.

*“In first year, we had clinical colloquiums, which is where we just had patients come in and just tell their perspective and their story and all of them being organic medical conditions, but a lot of us walked away from that and there was no assessment or anything linked to it; it was just for our own exposure and understanding from another person’s perspective, what it was like to live with that condition. And I loved them and I think I would have loved to have heard, at least from patients with particular perhaps… disorders like schizophrenia, like bipolar, talking about their experiences both in the health system, good and bad, and with different types of health professionals and perhaps how they like to have a consult.”* MD3, FG5

Participants provided suggestions to incorporate formal and informal contact-based interventions in the curriculum including both traditional lecture, tutorial and workshop formats where patients come into the classroom as teachers in addition to a range of clinical placements.

*“I think that having patients with lived experience teaching, saying like here’s how you interact with me when I’m psychotic, here are some of the things that you should do or not do or here’s what it feels like to be manic so that having patients with lived experience, doing the teaching, but also being involved in, like, what did they want their future healthcare workers to know about this and how do they want them to act.”* MD4, FG6

*“Rather than in hospital, I learned about it a lot more in the GP [General Practice] setting because the patients that I saw were well, and they could tell me their experience.”* MD3, FG3

This approach would require students to have the ability to interact with patients with mental health conditions on their clinical placements and not be asked by more senior physicians to stay out of the way. The challenges of this are explored further in the subtheme ‘Clinical application.’

#### Examples and exemplars

Participants discussed the importance of positive role models in learning how to complete their clinical duties with both efficiency and empathy. One participant described a toxicologist that conducted assessments in a compassionate and efficient manner in the ED, which left a lasting impression on them.

*“I think that one of the things that was most effective…was exposure to a toxicology consultant …who was incredibly empathetic and very respectful in all of his interactions with his patients. And I only spent one week on that team, but that exposure really did help me to see the more human aspect of addiction medicine.”* MD4, FG3

Several participants emphasised that witnessing both positive and negative examples of clinical care greatly impact their future approach as health care professionals. They emphasised the importance of opportunities for reflection and debrief after witnessing stigma to help resolve dissonance experienced by students. As noted by an MD4 student (FG3), students often feel conflicted about what to do when witnessing stigmatising attitudes and behaviours from other health care workers they engage with on clinical placements.

*"I think, as medical students, we come into the system with this different fresh perspective that people who have been in the system for a long time don't have. And I think that, if you see a patient, you'll often get the vibe from the treating team or the nursing staff, and you'll view the patient through that lens. And I've often found that sometimes it disconnects with what I felt."* MD4, FG3

One participant suggested providing opportunities for interprofessional learning to broaden students understanding of the broader social and cultural influences on patient care and outcomes. More specifically, they suggested learning from social workers. The participant wanted greater understanding of the social and cultural aspects that influence the patient’s care. Another noted that first year students are now placed in General Practice (GP) clinical settings and expressed that would provide the opportunity to build a foundation of learning by watching general practitioners’ interactions with patients with mental health conditions.

#### Clinical application

Clinical application is about the opportunity to apply the skills learned in the skills development phase. Participants generally agreed that they needed to have more opportunities to practice mental health skills in a clinical setting while being observed by others who can provide feedback and encourage reflective practice to enhance learning. Several participants gave examples of missing out on practicing and refining their communication and mental health clinical skills when being asked not to participate in mental health consultations.

*“When I’m at clinical placement, for example I’m in ED, a lot of the clinicians and consultants they don't really want us talking to mental health patients…They prefer us, oh, go look at the abdo pain guy, the chest pain, shortness of breath or something, I guess, they get us to avoid the topic of mental health.”* MD2, FG2

In three of the FGDs, participants spoke about learning how to do sensitive examinations (e.g., gynaecological examinations and discussion about bowel movements) where you learn the skills and practice them in a psychologically safe way before entering a clinical setting. They suggested this approach could be applied to learning about patients with mental health conditions.

*“… when I think back to at the beginning of second year, where we learned how to do things like sensitive examinations, I mean, I know that’s a more, like a very physical thing, but even that, just teaching us how to make sure we word things correctly, so we don’t accidentally trigger someone when we’re doing a sensitive exam.” MD3, FG4*

*“I found I learnt a lot from just watching other people do things and having a go doing it myself.”* MD3, FG4

#### Transform structural barriers

Many participants felt that the medical school itself has an important role to play in addressing the medical culture that can perpetuate both interpersonal stigma and self-stigma.

*“changing the system is hard and there's this hierarchy that's been built, which is so hard to shift. I think that's the real issue, and it's very hard for us as medical students to engage with that. It’s got to come from all levels, I think.”* MD4, FG3

While participants found it challenging to identify specific solutions to change medical culture, one suggestion was addressing the tone from leadership and senior educators when talking about mental health. For example, an MD2 participant (FG2) expressed,

*“…the [clinical] Dean at the time was, “Oh, yeah, we care about wellbeing, ha, ha.” So that’s an interesting one for both med students who would have mental health issues, and also sets the tone as how that’s valued in the clinical school, in that hospital, that’s a pretty poor way to start.”*

In contrast, an MD4 participant (FG6) noted that their dean of clinical school was more open about talking about personal experiences of difficult situations. As a result, the participant felt that they did not have to push their feelings aside after a challenging clinical situation. These participants illustrate the importance of consistency among leadership in addressing mental health stigma and how the approach taken can influence students' perceptions of initiatives. Participants acknowledged that several well-being initiatives have been implemented such as discussing the mental health of students in tutorials.

“*…I feel like this year or as well as last year, the med school, well, they renamed them PP [Professional Practice] tutes and I found they really emphasised the mental health of students and physicians so, I guess, there's an aspect of us recognising our own mental state, where we feel and what we’re comfortable with as well.”* MD2, FG2

A few specific strategies for the medical school to further support students to manage their own mental health were also suggested. These included enhanced communication and making the process of taking time off for reasons relating to mental health more streamlined. Many participants stressed that strategies to address students’ mental health should not be tokenistic.

When asked how students could be supported to speak up when witnessing stigma one participant suggested workshops to *“equip us with skills that we can use to call out derogatory comments in the workplace”* (MD4, FG4) This could be part of learning graded assertiveness. Another participant noted that timing matters – *“when the stigma shows up it’s perpetuated…if you don't really deal with it”* (MD3, FG2). So, participants need to be equipped and supported to speak up when the situations arise.

Participants did not provide any solutions to addressing the professional stereotypes that are often linked to psychiatry.

## Discussion

This study provided insight into where stigma exists in medical education and how medical students could be supported to prevent negative attitudes and behaviours from forming to improve patient care of people with mental health conditions. The six FGDs elucidated that stigma is pervasive throughout the medical culture including in the classroom and clinical settings. The FGDs illustrated that no one magic bullet exists, but a set of solutions emerged.

The participants noted that stigma could show up anywhere. Furthermore, several examples of where stigma presents are outside the ‘classroom,’ therefore medical educators cannot control these situations. However, educators can help build medical students’ stigma resistance so that when they do inevitably encounter stigma in clinical settings, they might be better equipped to resist and not let it impact their attitudes or behaviours as clinicians. For example, building stigma resistance could provide students with a skillset to protect against negative clinical role models*.* Or it could assist students with speaking up against stigma which is important for patient safety [35]. Researchers primarily apply the concept of stigma resistance to the experience of self-stigma (e.g., [36–39]. Boyd Ritsher et al., [39] defines stigma resistance as the capacity to counteract, resist or remain unaffected by mental health-related stigma. In this study, the solutions suggested by the participants lend themselves to extending the concept of stigma resistance towards interpersonal stigma. The participants provided several examples of skills to buffer against stigma, such as communication skills to speak up when witnessing stigma and exposure to different types of mental health conditions and patients in different parts of their recovery. Teaching stigma resistance through the techniques identified by study participants could enable medical students to respond not just to stigmatising situations when they arise, but also to anticipate them and adjust accordingly before the situation happens [40]. Ultimately, building stigma resistance could enhance care towards patients with mental health conditions and hopefully improve patient outcomes.

Many of the solutions identified in the ReSPECT model align with existing literature on stigma reduction (e.g., [16, 22, 23, 25]) but, when combined, have the potential to create greater resistance than just a single approach. Integration of interventions woven throughout the curriculum provides an opportunity to build a sustained skill set for stigma resistance over multiple years. Furthermore, it would also allow students to engage with mental health education before contact in clinical settings which evidence strongly shows more effectively reduces stigma [41]. Our findings align with the research that indicates multi-modal, more intensive approaches better address stigma than one-off short-term solutions [22, 42]. However, more longitudinal studies measuring attitudes and behavioural intentions are needed. This approach could help address the gap in the literature.

Building stigma resistance into the curriculum would signal to medical students the value of acquiring this skillset. A one-off intervention has the potential to seem tokenistic. As some of the participants noted, making these activities mandatory also signals to students that the school prioritises mental health. To them, mandatory and integrated learning activities indicate how they should focus their efforts and what is essential to their success as a future physician. More importantly it also seems to signal to them what the medical school and educators value.

Furthermore, framing ReSPECT interventions as stigma resistance versus anti-stigma could reframe the narrative and make these programs more palatable among health professionals and medical educators. Instead of blaming health professionals, it is another skill set that can help medical students provide better patient care. Although outside the scope of this study, self-stigma among health professionals, including medical students, is a widespread challenge [43]. Therefore, the added benefit of building stigma resistance is that it could also positively impact the self-stigma experienced by many medical students. Finally, given that the participants provided many examples of witnessing stigmatising behaviour from senior teachers and clinicians, an approach to building stigma resistance could be applied to educators as well.

### Strength and limitations

Our study is the first study to qualitatively explore how we could embed stigma reduction into the medical curriculum in Australia. Through FGDs and inductive coding, we were able to explore and identify suggestions made by students for students. Participants of different academic years, age, gender and clinical school were included to ensure a range of opinions and experiences.

The major limitation is that FGDs were conducted within a single institution and therefore some themes presented could be specific to the institution. Logistically, it was not possible to include all University of Melbourne medical students so that aspect could have introduced possible bias. For example, participation was voluntary and could have resulted in self-selection with those who have a particular interest in mental health-related stigma choosing to participate. As such, the sample is subject to possible bias and may not be representative of the cohort overall. However, many of the suggestions mirror what works in the stigma intervention literature.

Another limitation is that we only spoke with medical students and not medical educators. As such, it is possible that some of these learning activities do exist within the curriculum. However, what this paper illustrates is that there is a potential disconnect between learning intention and what the students perceive. Finally, this study is limited to the suggestions of medical students, nevertheless, the ReSPECT model could be applicable to all health professionals.

## Conclusions

Our study identified three main areas where mental health-related stigma presents for students in medical school – (1) through unpreparedness in dealing with patients with mental health conditions (2) noticing mentors expressing stigma and (3) through the culture of medicine. The results of our study also provide a model for building stigma resistance among medical students from the perspective of medical students themselves. The six techniques of the ReSPECT model – (1) reflection (2) skills building (3) patient experiences (4) examples and exemplars (5) clinical application and (6) transforming structural barriers provide promising ways for medical educators to integrate stigma resistance throughout the curriculum from year one to better equip students with the potential to reduce interpersonal stigma and perhaps self-stigma as well.

## Data Availability

The data are not publicly available due to privacy or ethical restrictions. However, you can contact the corresponding author to request anonoymous data.

## Abbreviations

COREQ: Consolidated Criteria for Reporting Qualitative Research
DSM-5: Diagnostic and Statistical Manual of Mental Disorders
ED: Emergency Department
FG: Focus group
FGD: Focus group discussion
GP: General Practice
MD2: Second year medical student
MD3: Third year medical student
MD4: Fourth (final) year medical student
UoM: University of Melbourne
